# Prevalence and risk factors of gastrointestinal parasitic infections in goats in low-input low-output farming systems in Zimbabwe

**DOI:** 10.1016/j.smallrumres.2016.09.005

**Published:** 2016-10

**Authors:** P.I. Zvinorova, T.E. Halimani, F.C. Muchadeyi, O. Matika, V. Riggio, K. Dzama

**Affiliations:** a Department of Animal Sciences, University of Stellenbosch, Private Bag X1, Matieland 7602, South Africa; b Department of Para-Clinical Veterinary Studies, University of Zimbabwe, P. O. MP167, Mt. Pleasant, Harare, Zimbabwe; c Department of Animal Science, University of Zimbabwe, P. O. MP167, Mt. Pleasant, Harare, Zimbabwe; d Biotechnology Platform, Agriculture Research Council Private Bag X5, Onderstepoort 0110, South Africa; e The Roslin Station and R(D)SVS, University of Edinburgh, Easter Bush, Midlothian EH25 9RG, Scotland, UK

**Keywords:** Faecal floatation, Gastrointestinal tract, Helminth, Coccidian, Risk assessment

## Abstract

A longitudinal study was conducted in low-input low-output farming systems to determine the prevalence of gastrointestinal parasitic infections in different age groups, sex and associated risk factors in goats. A total of 580 indigenous goats were randomly selected in areas representing the five agro-ecological regions of Zimbabwe in the dry and wet seasons. Blood and faecal samples were collected from each animal and egg/oocyst per gram of faeces (epg/opg), larval culture, and packed cell volumes (PCV) were determined. Factors affecting parasitic infections were evaluated. Highest prevalence was determined for *Eimeria* oocysts (43%), strongyles (31%) and lower levels in trematodes and cestodes. Parasites identified were *Haemonchus, Strongyloides* and *Oesophagostomum.* Area, season, sex and age significantly influenced patterns of gastrointestinal infections (*P* *<* 0.05). Cannonical correlations indicated that parasite species composition varied by area and impacts of risk factors also differed. Risk of infection was very high for goats sampled in Natural regions (NR) I, II, III (OR = 6.6–8.2; *P* < 0.05) as compared to those in NR IV and V. Highest helminths and *Eimeria* infections were observed in the wet vs. dry season (*P* *<* 0.05). Young animals were more susceptible to parasitic infections (*P* *<* 0.05). Prevalence was higher in males than females, with odds of infection for males being almost three times to that for females (*P* *<* 0.0001). Knowledge concerning gastrointestinal helminth biology and epidemiological infection patterns caused by these parasites is essential in the development of appropriate control strategies and this has a potential to reduce production losses.

## Highlights

• High prevalence for *Haemonchus* and *Eimeria* spp.
• Site, season, sex and age significantly influenced gastrointestinal infections.
• Highest level of infections in the wet season, in males and in young animals.
• Parasite species composition and risk factors also varied by area.

## 1 Introduction

Goats make important contributions to human livelihoods in developing economies, since they are extremely hardy animals that can survive and reproduce under extremely high temperatures and low humidity with minimum available feed. Of the approximately 1 billion world goat population, 56 and 30% are located in Asia and Africa respectively (FAO, 2015). The majority of the goats in Zimbabwe are owned by smallholder farmers in mixed crop-livestock systems (Rooyen and Tui, 2009). In this farming system, goats are increasingly used to augment cash income and enhance food security, thus serve as an important component in the household’s livelihood strategies. Socio-economic importance is attached to goat ownership such that, in some instances they may be the only realisable wealth of a rural household (Nwosu et al., 2007). In addition to that, goats have other functions such as provision of manure, cultural roles, thus playing a significant role in livelihoods.

Gastrointestinal nematode infections (GIN) are the main prevalent parasitic diseases affecting small ruminant productivity worldwide, especially in tropics and sub-tropics (Torres-Acosta and Hoste, 2008, Calvete et al., 2014). Globally the most common nematode species known to affect small ruminants are *Haemonchus contortus*, *Trichostrongylus colubriformis*, *Teladorsagia circumcincta* and some species such as *Nematodirus* spp, which are not found in sub-Saharan Africa (Bishop and Morris, 2007).

Large numbers of internal parasites and their prevalence have been documented in different studies of goats including Zimbabwe (Pandey et al., 1994), Namibia (Kumba et al., 2003), Nigeria (Nwosu et al., 2007), Kenya (Odoi et al., 2007), Ethiopia (Sissay et al., 2007), South Africa (Gwaze et al., 2009), Cameroon (Ntonifor et al., 2013); Tanzania (Sharma and Mandal, 2013) and others. The most common nematode genera detected in mixed infections in these studies were *Haemonchus, Trichostrongyles, Strongyloides, Trichuris, Bunostomum, Oesophagostomum, Cooperia, Nematodirus* spp (Badaso and Addis, 2015). Trichostrongyle nematodes are considered among the most pathogenic and economically important parasites of small ruminants (Jurasek et al., 2010) and knowledge of these species is important for preventing and managing parasitic diseases.

Gastrointestinal parasitism is associated with economic losses, lowered productivity, reduced animal performance (Badran et al., 2012), mortality and morbidity (Negasi et al., 2012). In general, severe GIN pathogenesis has been attributed to the migration of the infective larvae after ingestion rather than the adult worms in the gut (Dube et al., 2002). According to Risso et al. (2015), goats infected with internal parasites show a rough dull-coat, weakness, diarrhea, apathy, tail rubbing, signs of hypo-proteinaemia, submandibular oedema (bottle jaw), loss of appetite and weight loss. In addition, some trichostrongyle nematodes cause anaemia due to their ability to remove red blood cells as well as proteins, which can lead to ill-thrift in animals.

In addition to gastrointestinal nematodes, coccidiosis (especially *Eimeria* species) has also been known to infect livestock in Zimbabwe, having moderate to high pathogenic effects (Radfar et al., 2011). However, co-infection with other trichostrongyle nematodes is making diagnosis of clinical coccidiosis difficult (Chhabra and Pandey, 1991, Zainalabidin et al., 2015).

Various risk factors play an important role in the onset of GIN infections, due to host and environment. Environmental factors include agro-ecological conditions, animal husbandry practices such as housing system, deworming intervals and pasture management (Ratanapob et al., 2012); these largely determine the type, incidence and severity of various parasitic diseases (Badran et al., 2012). Other risk factors such as the host species, sex of the animal, age, body condition and breed/genotype (Badaso and Addis, 2015), parasite species and intensity of the worm population, have an effect on the development of gastrointestinal parasitic infections (Tariq et al., 2010).

Limited area-specific studies conducted in Zimbabwe (Pandey et al., 1994, Matika et al., 2003) have generated limited information on gastrointestinal parasite prevalence in the different agro-ecological regions and associated risk factors to parasite infection. Knowledge on the prevalence, specific composition of the gastrointestinal fauna can provide baseline information which can be used to control parasite infections. The specific objectives of the study were to: i) characterise GINs present in diverse farming systems,; ii) determine level of prevalence of the parasites considered; and iii) evaluate risk factors on parasite infections in goats reared in low-input, low-output systems in Zimbabwe.

## 2 Materials and methods

### 2.1 Study area and data collection

The study was conducted between November 2014 and June 2015 in low-input low-output farming systems in five districts of Zimbabwe: Chipinge, Shurugwi, Binga, Tsholotsho and Matobo, representing the five agro-ecological regions. Table 1 shows a description of the study districts.

### 2.2 Animal management

The animals from Chipinge, Shurugwi, Binga, Tsholotsho and Matobo were owned by smallholder farmers who had small flock sizes, ranging from 1 to 10. Animals from these areas were maintained under extensive management systems, where they foraged in farm land or in communal pastures during the day with minimum supplementation and kraaled during the night. In these areas, veterinary care was low to non-existent, with goats not treated/dewormed. Animals mated indiscriminately in communal grazing areas. Goats in these areas had contact with other animal species such as cattle and sheep in the communal grazing areas.

The animals at Matopos Research Station (in the district of Matobo) were managed semi-intensively. The goats foraged on the Research Station open rangeland throughout the year with some rotation in the paddocks during the day, minimum supplementation (1 kg of prepared meal of forage legumes + maize per animal) and penned at night. All animals were treated with an acaricide weekly during the wet season and fortnightly during the dry season to control ticks and tick-borne diseases. Ivermectin and Closantel were used routinely to control for gastrointestinal parasites. Mating was done yearly from June to August, with each buck mated with 25–30 does.

### 2.3 Animal ethical clearance

Animal use ethical clearance was approved by the Animal Ethics sub-committee of the Department of Livestock and Veterinary Services, Zimbabwe, according to the international standards of animals use in research; clearance certificate number 001/15/Animal.

### 2.4 Study animals

Sample size was determined using the equation ***n*** = 1.96^2^***pq/L***^2^ (Thrusfield, 1997), where ***n*** = sample size, ***p*** = expected prevalence, ***q*** = 1 ***− p*** and ***L*** = limits of error on the prevalence (absolute precision at 95% confidence interval). The expected prevalence was estimated at 80% in the communal areas. In addition, a 10% allowance for non-response in the communal herds was made, giving a total sample size of 270 goats. This led to goats of different ages being sampled depending on availability per farm. On the other hand, all the animals at Matopos Research Station were sampled, resulting in 310 additional animals of different age classes as indicated in Table 2.

The Mashona and Tonga goats are small, compact and hardy indigenous breeds. According to Mason and Maule (1960), these are prototypes of the Small East African goats, with mature body mass of 25–30 kg. The Matabele type goats are bigger than the Mashona, with mature body mass ranging from 40–65 kg for males and 30–45 kg for females.

### 2.5 Sample collection, examination and culture

Faecal and blood samples were collected directly from the rectum and jugular veins into airtight containers and EDTA vacutainer tubes, respectively. Sample collection was conducted over two different seasons targeting the dry (late April–early October) and wet (late October–early April) seasons from 2014 to 2015. Sampling was conducted in January, June and July (Tsholotsho); February, June and July (Shurugwi); April, June and October (Chipinge), February, May and November (Binga); January, May and September (Matobo); January, May, July and September (Matopos Research Station), with a total of 1872 records from 580 animals collected. Rainfall and temperature for the different areas was obtained from the Meteorological department.

Faecal egg counts (FEC) were determined by the modified McMaster technique, using floatation methods for nematodes, cestodes, and using sedimentation methods for trematodes (MAFF, 1986). Distinguishable nematode eggs (*Nematodirus and Trichuris*), trematode and cestode eggs were identified directly. Faecal cultures were prepared by incubating 2–3 g of faeces at 26–28 °C for 7 days at 80% humidity after which infective larvae were collected using a modified Baerman technique as described by Roberts and O'sullivan (1950). Identification of 3rd stage larvae of nematodes was only at the genus level according to Van Wyk et al. (2004). Another indicator of parasitic infestation assessed was packed cell volumes (PCV), using the capillary micro-hematocrit centrifuge method (Bull, 2000). PCV is usually associated with cases of helminthiasis (Zainalabidin et al., 2015), especially *H. contortus*, which cause anaemia. To complement the information on the samples collected, a questionnaire was administered and information on management practices, farmer knowledge on internal parasites and methods of control, was also recorded.

### 2.6 Statistical analyses

Analyses were carried out with the Statistical Analysis System (SAS, 2011). Descriptive analysis was conducted on survey data. The traits analysed were FEC for nematodes and coccidiosis and PCV. FEC for all nematodes and coccidia were transformed through a base 10 logarithm, log_10_ (FEC + 25) to approximate a normal distribution. The data and the results were back-transformed by taking anti-logarithms and presented as geometric means (GFEC). All statistical tests for FEC were applied to the transformed data. Fixed effects were explored using PROC GLM procedure (SAS, 2011) using the model:

Y*_ijkl_* = μ + *S_i_* + T*_j_* + U*_k_* + A*_l_* + A * T*_lj_* + A * U*_lk_* + *ε_ijkl_*

where Y*_ijkl_* is the response variable of LFEC (Lstrongyles, L*Fasciola*, Lamphistomes, L*Trichuris, LStrongyloides*, L*Moniezia*) and LCOCCIDIA, μ is the population mean; S*_i_* is the effect of the *i*^th^ study area (Chipinge, Shurugwi, Binga, Tsholotsho and Matobo districts); T*_j_* is the effect of the *j*^th^ sex (male or female); U*_k_* is the effect of the *k*^th^ season (wet or dry); A*_l_* is the effect of the *l*^th^ age (1–7years); A * T*_lj_* and A * U*_lk_* are the interactions (age * season and age * sex); *ε_ijkl_* is the random residual effect. Pairwise comparisons were carried out using the PDIFF option in SAS (2011). An ordinal logistic regression was used to determine the odds of infection status of the different parasites using the PROC LOGISTIC procedure (SAS, 2011):

$$log\frac{p}{1\;\;-\;\;p}=\beta_{0}+\beta_{1}X_{1}+\beta_{2}X_{2}+\beta_{3}X_{3}\ldots \ldots \ldots \beta_{n}X_{n}+\varepsilon_{ijkl}$$

where ***p*** is the probability of experiencing GIN infections; [***p***/1 − ***p***] is the Odds ratio, which refers to the odds of experiencing GIN infections; _β0_ is the intercept; _β1_…_βn_ are the regression coefficients of predictors; *X_1_⋯X_n_* are the predictor variables (sex, age, area, month, breeds, availability of housing, supplementary feeding, veterinary services, farmer knowledge on GIN, parasite control method, use of anthelmintics, anthelmintic class used); ε is the random residual error distributed as N (0, 1 σ^2^_E_). The best model was then chosen using stepwise selection. Overall fit of the logistic regression models was assessed using the Hosmer-Lemeshow goodness-of-fit statistics. The CANCORR procedure was then used to assess the relationships between parasites and the effect of risk factors on parasites in different sites.

Prevalence was calculated as a percentage of ***d****/**n*** where ***d*** is the number of animals infected and ***n*** is the total number of animals examined through FEC.

## 3 Results

### 3.1 Survey descriptive statistics

Descriptive statistics for goats in the different areas was generated for the management and healthcare of animals. Goats relied on natural foraging for feed (98.4%), and the remainder were provided with nutritional supplements in addition to the natural pasture, throughout the year. Ninety-five percent of the farmers provided their goats with housing (kraals) and the remainder did not. Majority of the farmers in the communal areas (69.5%) has access to healthcare services i.e. government or private veterinary practitioners. Despite this, 57.9% of the farmer did not control for gastrointestinal parasitic infections. Farmers were examined on the general knowledge of parasitic infections and 62.8% did not have the knowledge.

### 3.2 Prevalence of helminths and coccidia in sampled goats

The L_3_ nematodes identified from faecal cultures of all animals, across all age groups were *Haemonchus* and *Oesophagostomum* in Chipinge, Matobo (communal and Research Station) and Tsholotsho. In Shurugwi parasites identified were *Haemonchus* and *Strongyloides*, while in Binga all faecal cultures were negative for any genera of nematodes. Mixed infections, comprising of 14% of the faecal cultures, were composed of *Haemonchus, Oesophagostomum* and *Strongyloides*, with cases of mixed infections highest in Chipinge (NR 1).

FEC were highly variable for the different areas as summarised in Table 3. Over the study period, majority of the animals had FECs of zeros for all the parasites, with 43 to 80% (dry–wet season) for *Strongyles* being documented and 55–60% for coccidia (*Eimeria* species). Level of infection was low for all groups of parasites ranging from *Strongyles* (143.8 ± 14.87 epg), *Eimeria* spp (216.2 ± 21.44 opg) and even lower for the other species, ranging from 0.04 ± 0.00 to 6.0 ± 4.32 epg. The highest epg recorded for *Strongyles* was 370.2 ± 44.56 epg in the wet season and for *Eimeria* species in goats aged 1 (457.7 ± 82.31 epg).

The highest prevalence (43%) was observed for *Eimeria* spp., followed by nematodes (31%), trematodes (5%) and cestodes (0.4%). Prevalences for all parasites were generally low in the Research Station flock as compared to the communal areas (*P* < 0.05). Information from FEC was used in Table 4 to summarise the prevalence of different gastrointestinal parasite across study areas.

Prevalence levels were higher for youngest animals i.e. yearlings (76%) *vs.* oldest goats i.e. 7 years (38%). *Eimeria* infections were the most prevalent parasitic infection, followed by *Strongyles*, lastly infections from the remaining species in all age groups. The level of *Strongyles* and *Eimeria* infections were generally low in goats of all age groups, using the intensity scales (levels of infection) by Hansen and Perry (1994), also by Asha and Chebo (2015). The highest levels of *Eimeria* infection were among yearlings (457.7 ± 82.03 opg) and those aged 6 years (320.3 ± 146.7 opg). *Strongyles* infections were low, at 292.2 ± 134.7 epg, for six-year old goats and (129.5 ± 24.3–207.2 ± 46.1 epg) for 1–3 year-old goats. The overall prevalence of the total internal parasites was higher in males (77%) than in females (55%) as summarised in Table 5 (*P* < 0.05). Conversely, the prevalence of *Strongyloides* and *Moniezia* spp. infections was higher in females than in males.

**Fig. 1:**
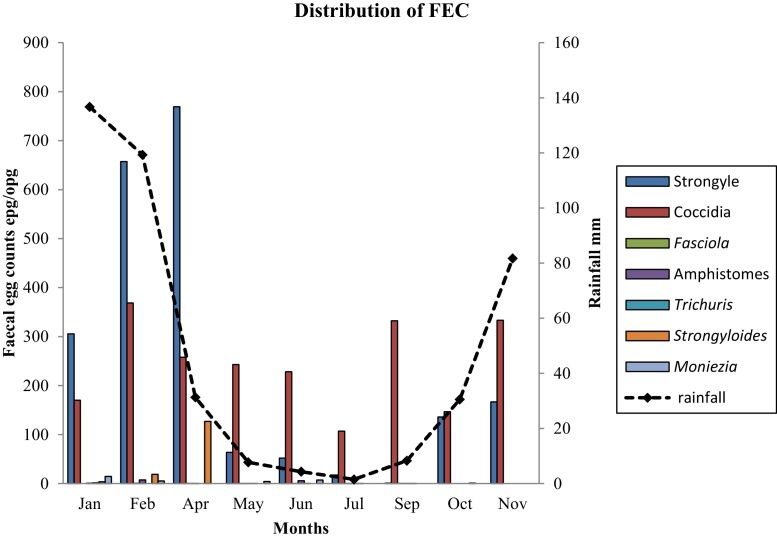
Rainfall patterns and mean monthly faecal egg counts for goats in all agro-ecological regions in Zimbabwe (There was no sampling in March, August and December), FECs for *Fasciola* spp., Amphistomes*, Trichuris* spp.*, Moniezia* spp. were very low, hence the shape of the graph.

Prevalence of infection was high in wet (64%) vs. dry season (36%). In addition to that, the means (epg/opg) were calculated to assess the distribution and level of infection by month (Fig. 1). *Strongyles* and coccidian distribution followed the rainfall patterns in Zimbabwe with high counts obtained in hot-wet as compared to the cold-dry months.

Least squares means by season and sex for PCV, LFEC, LCOCCIDIA and GFEC at different ages are presented in Table 6. PCV, LFEC, GFEC were high for wet vs. dry season, males vs. females for all age groups and these were significantly different (*P* < 0.05) among each other and between their interactions (age * season and age * sex). Three percent of the animals had low PCV of less than 20%. Phenotypic correlation between *Strongyles* and PCV was relatively very weak and non-significant (r = 0.003; *P* > 0.05). In our study the levels of PCV were low and non-significant, this can be explained by that only a few GIN cause anaemia and not all of them, the most common being *Haemonchus*. Fixed effects and their interactions were also tested for their effect on different parasites using the model stated. Area of sampling had significant effects (*P* < 0.05) on all parasites except L*Trichuris* and L*Moniezia*. Sex, age and the interaction of season*age had significant effects on L*Strongyles* and LCoccidia (*P* < 0.05). Season had significant effects on LStrongyles, LAmphistomes and *LStrongyloides* (*P* < 0.05). The interaction of sex*age had no effect on parasitic infections.

### 3.3 Risk factors associated with gastrointestinal parasite infection

Factors affecting gastrointestinal parasitic infections are summarised in Table 7. Odds ratios indicated that area of sampling and age of animal had the highest effect on parasite infection. In addition to area of sampling and age; season and sex of goats also had significant effects on the distribution of parasites (*P* < 0.05). Odds ratios for the effect of month were generally low, but the highest/peak infection were in February (OR = 0.68) and the lowest from April to October (OR = 0.14–0.22), which indicates the start of the dry season. Goats sampled from Chipinge and Shurugwi (NR I, II and III) districts had the highest risk of parasitic infection (OR = 6.6–8.2; *P* < 0.05) as compared to those from dry and hot Tsholotsho, Binga and Matobo districts (NR IV and V). The risk of infection was highest at the extreme ages 1, 6 years; moderate at 2, 3, 5 years and lowest at 4 years (*P* *<* 0.05). The odds for males being infected with intestinal parasites were 2.8 higher than for females (*P* *<* 0.0001).

### 3.4 Association of risk factors with parasitic infections in different areas

Canonical analyses were used to further explore the parasite patterns and the impact of factors in different areas. Eigenvectors indicated that *Eimeria* and *Strongyles* were the most common parasites across the areas. In Binga, parasitic infections of *Strongyles*, *Eimeria* and amphistomes were the most common. Moderate to high correlations between breed (r = 0.50), month (r = 0.50) and availability of supplementary feed (r = 0.82) were associated with *Eimeria, Strongyles* and amphistomes infections. Comparing risk factors indicated that increasing supplementary feeding reduced the need for administering anthelmintic control (r = −0.75). Infections in Chipinge included those from *Eimeria* and *Trichuris*. Low infections in *Eimeria* were associated with a decrease in age (r = −0.36) and lack of parasitic control (r = 0.31). Results indicated that the absence of anthelmintic treatment had low correlations (r = −0.24) with *Trichuris* infections. The use of SCL and ML class of anthelmintics was highly negatively associated with *Trichuris* infections (r = −0.91). *Eimeria* and *Strongyloides* infections were most common in Matobo. The absence of veterinary services was associated with *Eimeria* infections (r = −0.46) and the effect of month had a high negative relationship with *Strongyloides* infection. Common parasitic infections in Shurugwi included *Strongyles*, amphistomes and *Moniezia*. In this area, month had strong negative relationship with *Strongyles* and amphistomes infection. Low *Moniezia* infections (r = 0.46) were associated with use of indigenous breeds, while use of the same breeds showed an increase in amphistomes infections. Several risk factors were responsible for *Strongyles* and *Eimeria* infections in Tsholotsho. Low infections in *Strongyles* and *Moniezia* were positively associated with month, age, availability of supplementary feeds, with correlations ranging from 0.36 − 0.7, the converse of that was reported for *Eimeria.* The absence of anthelmintic treatment (r = −0.46) favoured a decrease in *Strongyles* and *Moniezia* infection while *Eimeria* n increased. Common parasitic infections in the Research Station flock included *Strongyles*, *Strongyloides Fasciola* and *Trichuris*. The availability of housing, supplementary feeding, veterinary services, had strong negative correlations (r = −1) with the increase in all parasitic infections in the flock. Sex had a moderate effect (r = 0.48) on *Strongyles* and amphistomes infections, which were high in males. *Fasciola* infections were high in females and lack of anthelmintic use was associated with low *Fasciola* infections. Factors affecting parasitic infections differed in different areas.

## 4 Discussion

Our study identified goat internal parasite species in different geographical areas which were differentiated by annual rainfalls and vegetation patterns. We also quantified the prevalence of these parasites using FEC obtained in goats of different ages, in two seasons across study areas. The parasite prevalence in our study was similar to the one reported by Odoi et al. (2007) and Shija et al. (2014). In these studies *Strongyles* and *Eimeira* species were the most prevalent parasites and prevalences were high in the rainy season. In our study, high infections with *Strongyles* and *Eimeira* species could be explained by the environment in which the goats were being reared, and also by poor animal management. Goats were reared in mixed crop-livestock systems, where a few numbers of goats were herded together in the same area during the dry and wet seasons. This results in high rates of parasitic infection due to possibilities of re-infection in contaminated pastures. However lower infections in the Research Station flock could be explained by improved management in terms of housing, feeding and healthcare. The other factor could be that goats had access to browse forage such *Acacia* bush which is dominant in the Research Station farm.

The most prevalent nematodes were *Strongyles*, with *Haemonchus* being the most common one. Previous studies on the epidemiology of gastrointestinal helminths have also reported *Haemonchus* as the most important nematode (Vassilev, 1995, Tsotetsi and Mbati, 2003, Bakunzi et al., 2013, Ntonifor et al., 2013). Its higher prevalence could be due to that adult females are capable of producing thousands of eggs per day, which can lead to rapid larval pasture contamination and associated outbreaks of haemonchosis (Roeber et al., 2013). There is also a role for climatic conditions since the parasite has high biotic potential and its pathogenicity which escalates the problem in humid tropics and subtropics (Waller and Chandrawathani, 2005). Another downside of *Haemonchus contortus* is its great ability to develop resistance to anthelmintic drugs (Kotze and Prichard, 2016), which poses a problem in terms of control.

The low prevalence of trematodes (amphistomes) was observed across the areas. However, high prevalence of amphistomes was noted in Shurugwi, this could be attributed to type of animal management and the weather patterns of this region, which has ambient temperatures and annual rainfall recorded in the sampling period of 20.3 °C and 675 mm respectively. These results recorded of high trematodes are in accordance with reports by (Godara et al., 2014). Previous studies conducted in Shurugwi by Dube et al. (2002) identified Calicophoron and *Paramphistomum clavula* as the dominant trematodes. Results for *Fasciola* were as low as those reported by Khanjari et al. (2014). According to these authors, for the development of the intermediate host, temperature (>9.5 °C), rainfall and soil moisture are also important factors influencing the development of the parasite from egg to miracidium. However, infections may have been low in goats due to their browsing/foraging behavior, which minimizes chances of ingesting the metacercaria which are found on plants closer to the ground.

The only cestode observed in the study area was *Moniezia* spp. The occurrence of this parasite in the tropics is associated with the ingestion of oribatid mites infected with larvocysts of *Moniezia s*pp. (Diop et al., 2015). In addition to *Haemonchus, Trichostrongylus, Oesophagostomum*, *Trichuris* and *Strongyloides* have been recorded in other studies (Tsotetsi and Mbati, 2003, Ayaz et al., 2013, Tsotetsi et al., 2013).

It has been proposed that the prevalence of different species reported in literature can be explained by different geographical distribution, host factors and climatic conditions required for the development of free-living stages of different nematodes. In this study, only *Strongyle* eggs and *Eimeria* oocysts (Fig. 1) showed a definite seasonal prevalence that corresponded to the rainfall patterns, similar to those previously reported by Nwosu et al. (2007) and Singh et al. (2013). We observed an increase in FEC and infection from October with a peak in April, which falls into the wet season. These observations were also reported by Pandey et al. (1994) in Zimbabwe. Infections continued into the dry season though the level of infection was low; this could be explained by the continued presence of worms in the host even during the dry season, when environmental conditions preclude the development and survival of their pre-parasitic stages. This observation indicates that rainfall and temperatures play a significant role in the epidemiology of gastrointestinal parasites as reported by Regassa et al. (2006). According to Magona and Musisi (2002) under satisfactory environmental conditions in the wet season, L_3_ larva of *Haemonchus contortus* and other *Strongyles* that infect goats reach infective stages within 46 days; this supports increased FEC in the wet *vs*. dry periods.

The findings that *Eimeira* spp. infestation was higher in young goats compared to adult goats, in terms of both prevalence and level/intensity of infection, and these findings were consistent with reports by Gwaze et al. (2009). Most of the goats that had helminths infection also harboured coccidian oocysts; the same results were obtained by Waruiru et al. (2000). According to Sharma and Mandal (2013), this may be associated to landholdings in the households, which directly determine the level of livestock management like sanitation, better living space and nutrition. In the current study, management in the smallholder system was characterized by overcrowding in small kraals/housing, poor nutrition, frequent exposure to communal grazing that have been contaminated and non-existent health control measures in place as also reported by Lone et al. (2012).

The observed high number of animals with zero FECs is in line with the expectation (Odoi et al., 2007). In addition to that there were low levels of infection; this could be due to that local breeds have acquired strong immunity to infection of GIT parasites due to recurrent infections. According to Baker et al. (1998), most goat breeds that are highly resistant to parasite infection are found in the tropics, but they lack desirable productivity traits.

Factors affecting the FEC reported in the study such as study area, season, age were the same as those reported by Sissay et al. (2007), with the exception of sex and time of sampling. In addition to that, age-wise prevalence revealed significant differences between age groups, with young animals being more susceptible and having higher FEC than the adult animals. These findings were in agreement with previous work (Tariq et al., 2010, Lone et al., 2012, Ayaz et al., 2013) in which naïve animals were reported to be more susceptible to infections. The protective effect in older animals is therefore, attributed to acquired immunity through frequent exposure (Odoi et al., 2007).

We observed that males of all ages were more susceptible than females as indicated in Table 6. These findings were similar to those by Tariq et al. (2008), Ayaz et al. (2013), Nabi et al. (2014), as well as Badaso and Addis (2015), who attributed this to the genetic predisposition and differential susceptibility owing to hormonal control. In these studies young and adults were considered. The results from this study were contrary to the findings of Emiru et al. (2013) and Vieira et al. (2014) in Ethiopia and Brazil, where females were more susceptible to parasite infection than males. This was attributed to lowered resistance of female animals due to their reproductive events and insufficient/unbalanced diet against higher needs. There were different parasitic infections in different areas. Impact of various risk factors was assessed and these varied across area on different parasites. Each parasite species was found in at least two different areas. Of all the factors assessed, the effects of month, age, supplementary feeding and anthelmintic use were the most dominant factors affecting parasitic infections. These differences can be explained by varying environmental and animal factors and also management systems in these areas.

Levels of infection for the indigenous goats were low, using the intensity scales by Hansen and Perry (1994), also by Asha and Chebo (2015). These findings were consistent with reports from Odoi et al. (2007). Low intensities could also be associated with the vegetation type that the goats were exposed to, in different areas as summarised in Table 1. Access to trees or shrubs with high levels of tannins e.g. *Acacia* has the ability to reduce levels of infection. Evidence of the anthelmintic properties of plants and plant-extracts is derived primarily from ethno-veterinary sources, most of which have been widely documented (Githiori et al., 2006). In addition to that, low infection levels can be attributed to the individual host’s ability to deter infection, or tolerate certain levels of infection without showing susceptibility. The impact of nematode infection was not assessed, but the prevalence determined in this study may be regarded as a problem affecting productivity of animals especially in mixed livestock-crop farming systems where farmers do not provide nutritional supplements, or invest in acquiring drugs for controlling parasites (Kumba et al., 2003).

## 5 Conclusion

The results from the study indicated that prevalence was high for *Strongyles* and *Eimeria* oocysts, with *Haemonchus* being the most common parasite identified. Despite this, a lower percentage (3%) of these animals was anaemic. The study identified area, sex, age and month as the most relevant risk factors for the development of gastrointestinal parasites across agro-ecological regions. Furthermore, the effect of site was explored for impact of different risk factors on parasitic infections and common parasite species and risk factors differed with area. Knowledge on these gastrointestinal helminths species and of the epidemiological parameters is important in the development of appropriate control strategies for the different areas. This has a potential to reduce production losses and improve rural livelihoods.

## Figures and Tables

**Table 1:** Agro-ecological zones/natural regions (NR) of Zimbabwe and vegetation.

NR	District	Rainfall (mm yr^−1^)	Temp (°C)	Altitude (m)	Vegetation
I	Chipinge	>1000	18.2	>1600	**Mountain grassveld:***Themeda, Loudetia, Andropogon, Monocymbium, Eragrostis* spp. Shrubs*: Senecio* spp.
II	Chipinge	750–1000	18.2	1200–1675	**Hyparrhenia tall grassveld:***Hyparrhenia, Hyperthelia, Heteropogon, Brachiaria, Digitaria, Eragrostis, Andropogon* spp. Shrubs *Terminalia, Burkea, Combretum, Acacia* spp.
III	Shurugwi	650–800	17.6	>1200	**Hyparrhenia and Eragrostis veld**: *Eragrostis*, *Heteropogon, Themeda, Cymbopogon, Hyparrhenia* spp. Shrubs *Acacia, Brachystegia, Julbernardia* spp.
IV	Binga	450–650	25.3	450–1050	**Eragrostisveld***: Eragrostis Schizachyrium, Heteropogon, Schmidtia, Pogonarthria, Brachiaria, Urochloa, Digitaria, Enneapogon, Aristida* spp. Shrubs: *Terminalia, Combretum*, Acacia, *Commiphora*, *Colophospermum, Grewia*, *Brachystegia, Enneapogon* spp.
IV	Tsholotsho	450–650	20.9	450–1050	**Eragrostisveld***: Eragrostis Schizachyrium, Heteropogon, Schmidtia, Pogonarthria, Brachiaria, Urochloa, Digitaria, Enneapogon, Aristida* spp. Shrubs: *Terminalia, Combretum*, Acacia, *Commiphora*, *Colophospermum, Grewia*, *Brachystegia, Enneapogon* spp.
V	Matobo	<450	19.9	900–1200	**Aristida and Eragrostis veld:***Aristida, Digitaria, Triraphis, Heteropogon, Eragrostis*, *Panicum, Baikiaea* spp. Shrubs: *Colophopsermum, Pterocarpus, Julbernardia, Brachystegia, Burkea, Terminalia, Guibourtia, Combretum* spp.

Modified from (Vincent et al., 1960) and (Gambiza and Nyama, 2000).

**Table 2:** Summary of households sampled across geographical locations.

NR	District/area	No. of sampled animals	aPredominant breed
I	Chipinge	30	Mashona
II	Chipinge	26	Mashona
III	Shurugwi	54	Mashona
IV	Binga	56	Tonga
IV	Tsholotsho	52	Matabele
V	Matopos Research Station	52	Matabele
V	Matobo	310	Matabele
Total	5	580	

a In each of the communal areas farmers kept predominant breeds together with crossbreds.

**Table 3:** Summary statistics (mean ± SE, range) of gastrointestinal parasitic infections in goats in different areas in Zimbabwe.

Area		Strongyles	*Fasciola* spp.	Amphistomes	*Trichuris* spp.	*Strongyloides* spp.	*Moniezia* spp.	*Eimeria* spp.
Binga	aMean FEC (Range)	191.7 ± 45.91 (0–1950)	0.2 ± 0.18 (0–9)	3.5 ± 2.01 (0–90)	0	0	0	290.6 ± 54.66 (0–1500)
Chipinge	Mean FEC (Range)	314.4 ± 60.78 (0–7700)	0.04 ± 0.034 (0–6)	0.02 ± 0.01 (0–2)	0.31 ± 0.29 (0–50)	41.0 ± 34.64 (0–6000)	6.6 ± 6.10 (0–1050)	188.3 ± 28.44 (0–2500)
Matopo	Mean FEC (Range)	309.3 ± 43.21 (0–3600)	0	0.2 ± 0.10 (0–11)	0.6 ± 0.59 (0–75)	5.1 ± 4.20 (0–500)	0	103.0 ± 39.66 (0–4500)
Shurugwi	Mean FEC (Range)	277.8 ± 70.71 (0–8650)	0.2 ± 0.11 (0–11)	9.7 ± 5.14 (0–703)	0.4 ± 0.35 (0–50)	7.3 ± 5.15 (0–700)	2.0 ± 1.75 (0–250)	263.7 ± 43.88 (0–2950)
Tsholotsho	Mean FEC (Range)	114.0 ± 23.22 (0–2350)	0	0	0.4 ± 0.35 (0–50)	2.6 ± 1.50 (0–150)	28.1 ± 26.43 (0–3800)	247.8 ± 68.68 (0–9000)
Research station	Mean FEC (Range)	56.1 ± 8.25 (0–3800)	0.01 ± 0.005 (0–3)	0.2 ± 0.05 (0–34)	1.8 ± 1.00 (0–1000)	0	1.6 ± 1.00 (0–1000)	230.5 ± 25.62 (0–17450)

a Mean FEC: means were calculated on non-transformed faecal egg counts so as to observe the levels/intensities of infection.

**Table 4:** Prevalence (%) of gastrointestinal parasitic infections in goats in different areas in Zimbabwe.

Area	Binga	Chipinge	Matopo	Shurugwi	Tsholotsho	Research station
Strongyles	61.5	62	77.6	50.8	43	15.7
*Fasciola* spp.	5.1	1.3	0	4.9	0	0.9
Amphistomes	12.8	0.6	3.45	24	0	3.1
*Trichuris* spp.	0	0.6	0.9	0.8	0.8	0.6
*Strongyloides* spp.	0	2.5	2.6	1.6	2.3	0
*Moniezia* spp.	0	0.7	0	0	0.7	0.4
*Eimeria* spp.	56.4	51.2	26.7	53.3	39.1	40

All prevalences were calculated using faecal egg counts.

**Table 5:** Prevalence (%) for helminths and coccidian parasites by sex of goats in different areas in Zimbabwe.

Area	Sex	Strongyles	*Fasciola* spp.	Amphistomes	*Trichuris* spp.	*Strongyloides* spp.	*Moniezia* spp.	*Eimeria* spp.
Binga	Male	66.7	11.1	16.7	0	0	0	55.6
	Female	57.1	0	9.5	0	0	0	57.1

Chipinge	Male	63.4	0	0	0	2.4	0	48.8
	Female	61.5	1.7	0.85	0.9	2.6	0.9	52.1

Matopo	Male	80.7	0	5.3	0	0	0	24.6
	Female	74.6	0	1.7	1.7	5.1	0	28.8

Shurugwi	Male	54	2	18	2	2	0	56
	Female	48.6	6.9	28.1	0	1.4	0	51.4

Tsholotsho	Male	38.9	0	0	1.7	3.4	1.7	42.4
	Female	46.4	0	0	0	1.5	0	36.2

Research station	Male	27.3	1.8	6.7	1.4	0	0	53.9
	Female	8	0.2	0.7	0	0	0.7	30.7

**Table 6:** Least squares means ± S.E. by season and sex for different ages for packed red cell volume (PCV (%)) logarithm transformed faecal egg counts (LFEC) for helminths/coccidian oocysts and geometric mean of faecal egg counts (GFEC (EPG)).

Age/yrs	Trait	Season		Sex	
		Dry	Wet	Male	Female
1	PCV	25.7 ± 0.59	26.7 ± 0.77	24.9 ± 0.60	27.5 ± 0.71
	aLFEC	1.7 ± 0.03	2.1 ± 0.05	2.0 ± 0.03	1.9 ± 0.04
	LCOCCIDIA	2.2 ± 0.04	1.8 ± 0.05	2.1 ± 0.04	1.9 ± 0.05
	GFEC	94	203	223	153

2	PCV	27.2 ± 0.57	30.1 ± 0.86	27.4 ± 0.68	30.0 ± 0.69
	LFEC	1.8 ± 0.03	2.2 ± 0.05	2.0 ± 0.03	1.9 ± 0.04
	LCOCCIDIA	1.9 ± 0.04	1.8 ± 0.06	1.9 ± 0.05	1.8 ± 0.05
	GFEC	89	252	307	153

3	PCV	27.9 ± 0.58	28.0 ± 0.80	28.7 ± 0.82	27.3 ± 0.55
	LFEC	1.8 ± 0.03	2.1 ± 0.04	2.0 ± 0.04	1.9 ± 0.03
	LCOCCIDIA	1.8 ± 0.04	1.9 ±0.06	1.9 ± 0.05	1.8 ± 0.04
	GFEC	135	257	171	201

4	PCV	27.3 ± 0.60	28.5 ± 0.83	28.0 ± 0.82	27.9 ± 0.60
	LFEC	1.8 ± 0.03	1.9 ±0.06	2.1 ± 0.04	1.9 ± 0.03
	LCOCCIDIA	1.9 ± 0.04	1.9 ± 0.06	2.0 ± 0.06	1.8 ± 0.04
	GFEC	76	281	167	133

5	PCV	26.9 ± 0.90	29.5 ± 1.63	28.1 ± 1.65	28.2 ± 0.88
	LFEC	1.8 ± 0.04	1.9 ± 0.09	1.8 ± 0.09	1.8 ± 0.05
	LCOCCIDIA	1.8 ± 0.06	2.1 ± 0.12	2.1 ± 0.12	1.9 ± 0.06
	GFEC	69	277	57	127

6	PCV	25.7 ± 1.72	27.1 ± 2.32	26.7 ± 1.80	26.2 ± 2.23
	LFEC	1.8 ± 0.09	2.6 ± 0.12	2.4 ± 0.09	2.0 ± 0.12
	LCOCCIDIA	2.1 ± 0.12	1.7 ± 0.16	2.2 ± 0.13	1.6 ± 0.16
	GFEC	32	289	157	59

a Infection with all the investigated parasites except coccidian oocysts. LFEC includes all helminthes infection.

**Table 7:** Odds ratio estimates and confidence limits for fixed factors affecting gastrointestinal parasite infection.

Effect	Odds Ratio	95% Wald Confidence limit		Significance
		Lower limit	Upper limit	
Area	23.562	10.904	52.746	***
Sex	0.365	0.286	0.467	***
Age	9.001	4.195	19.709	*
Month	2.106	0.187	23.989	*

* *P* < 0.05.

*** *P* < 0.001.
